# A Case of Acute Motor and Sensory Axonal Neuropathy Secondary to SGN-LIV1A Therapy

**DOI:** 10.7759/cureus.30197

**Published:** 2022-10-11

**Authors:** Emily R Daigle, Adeel S Zubair, Jeffrey J Dewey

**Affiliations:** 1 Neurology, Yale School of Medicine, New Haven, USA

**Keywords:** physical medicine and rehabilitation (pm&r), rehabilitation, neuro-immunology, guillain- barré syndrome, electromyography (emg), clinical case report

## Abstract

Antibody-drug conjugate therapy is rarely associated with neurologic immune-related phenomena. In this case report, we present a patient on treatment with SGN-LIV1A antibody-drug conjugate for breast cancer who developed progressive asymmetric quadriparesis, more severe in the bilateral upper extremities. Acute motor and sensory axonal neuropathy (AMSAN), a sub-variant of Guillain-Barré syndrome, was diagnosed via electro-diagnostic studies. Serological studies were significant for vitamin B1, B2 and B6 deficiencies, and cerebrospinal fluid studies were significant for albuminocytologic dissociation. The patient was treated with intravenous immunoglobulin (IVIg), B complex supplementation, and aggressive physical therapy. There was recovery of muscle strength in all extremities over the course of three months. Our case explores the biologic response to treatment of experimental immunotherapy-induced AMSAN with intravenous immunoglobulin.

## Introduction

Guillain-Barré syndrome (GBS) affects 1-2/100,000 people worldwide, however, the axonal variants are rare in the United States, comprising only 6% of GBS cases [1]. Acute motor and sensory axonal neuropathy (AMSAN) is an axonal sub-variant of GBS, and is an acquired neuropathy caused by autoimmune reaction against epitopes in the axonal membrane. In North America, cases are classically preceded by Campylobacter jejuni infection, though are less commonly preceded by cytomegalovirus (CMV) or Epstein-Barr virus (EBV) [2], and rarely associated with certain cytotoxic medications such as vincristine [3]. The hallmark of AMSAN is the involvement of both dorsal and ventral nerve roots, as opposed to the other axonal subtype of GBS, acute motor axonal neuropathy (AMAN) which involves the ventral root only [4]. Clinically, it often presents with rapid onset of sensory symptoms and motor weakness with maximal symptom onset before four weeks. It is diagnosed via electromyographic and nerve conduction studies, which show normal motor conduction velocity and latency with decreased amplitude of compound muscle action potentials.

While other variants of GBS have been reported to be caused by immune checkpoint inhibitor therapy such as pembrolizumab [5-6], AMSAN has not. To our knowledge, AMSAN has never been reported in association with antibody-drug conjugate therapy. In the present case, we report a patient who developed AMSAN shortly after completing a course of SGN-LIV1A (ladiratuzumab vedotin), an antibody-drug conjugate.

## Case presentation

In this study, we report the case of a 62-year-old female with estrogen receptor (ER)/progesterone receptor (PR)/human epidermal growth factor receptor 2 (HER2) negative breast cancer metastatic to the brain who had previously completed a six-month course of pembrolizumab (an immune checkpoint inhibitor) and five months later was started on investigational immunotherapy with a three-month course of SGN-LIV1A (an antibody-drug conjugate). This therapy is composed of an antibody to LIV1A, a zinc transporter often highly expressed in breast cancer, and a microtubule inhibitor (monomethyl auristatin E) [7].

**Figure 1:**
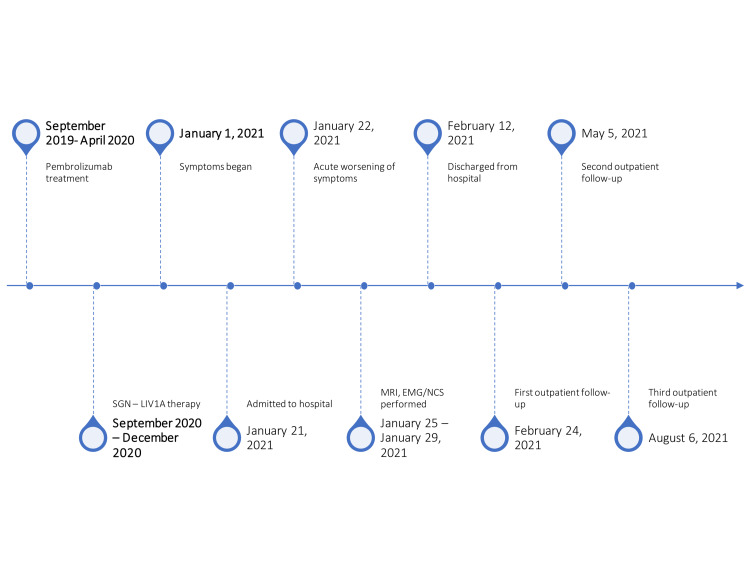
Timeline of Symptoms

The patient developed progressive generalized weakness and increased numbness/paresthesias in the fingers bilaterally over the course of two weeks, which acutely worsened during hospitalization for a deep venous thrombus/pulmonary embolism. Neurologic examination initially showed strength of deltoid 1/5 right and 4-/5 left, adduction at the shoulder 4-/5 bilaterally, biceps 1/5 right and 4-/5 left, triceps 4-/5 right and 4/5 left, hand grip 2/5 right and 4-/5 left, and dorsal interossei 1/5 right and 4-/5 left with 4/5 strength in the bilateral lower extremities. Reflexes were initially diminished but equal in the extremities throughout, apart from an absent Achilles reflex bilaterally. Sensation to light touch was subjectively decreased in the right upper extremity with a weak hand grip. The following day, motor strength decreased to 2/5 in the bilateral upper extremities (Figure 1).

**Figure 2:**
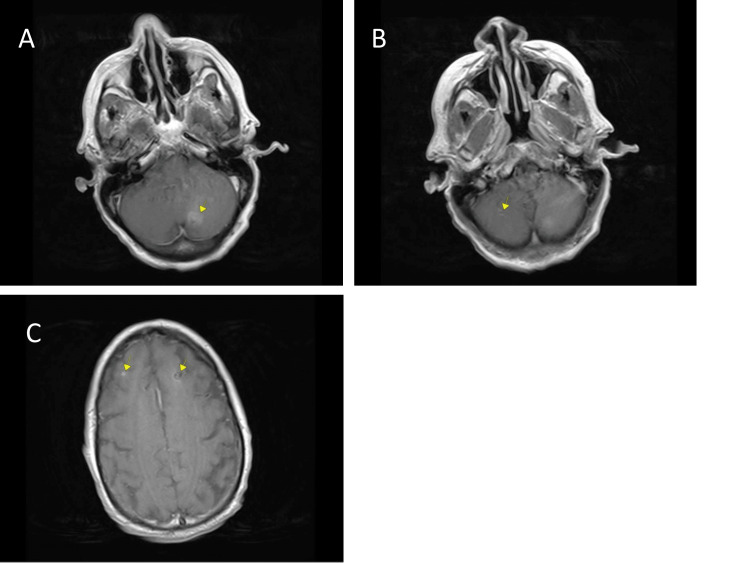
MRI Brain Axial T1 Post-Contrast A - 1.4 cm L cerebellar lesion; B - 3 mm R cerebellar lesion; C - 4 mm R anterior frontal lesion, and 1.1 cm L frontal lesion with hemorrhagic metastatic focus

MRI total spine (cervical, thoracic and lumbar) with and without contrast was performed and did not reveal any relevant lesions. MRI brain with and without contrast revealed new enhancing lesions in the bilateral cerebelli and right anterior frontal lobe, suggestive of metastasis (Figure 2). However, these cerebellar/frontal lesions did not explain the patient’s acute motor weakness and sensory symptoms, thus additional tests were ordered to determine the neurologic etiology of her symptoms. Cerebrospinal fluid analysis was significant for 2 WBC, protein 55 and glucose 84, consistent with albuminocytologic dissociation. Serological studies revealed undetectable vitamin B1 and B6, low levels of vitamin B2 (5.4 nmol/L) and a normal level of B12 (365 pg/mL). A sensory motor neuropathy panel was negative for autoantibodies to GM1, GD1a, GD1b and GQ1b. Nerve conduction studies revealed electrodiagnostic evidence of moderate-to-severe sensorimotor length-dependent polyneuropathy with predominantly axonal features (Tables 1, 2). On electromyography, the pattern of spontaneous activity and motor unit action potential morphology in the distal muscles was consistent with a subacute, not chronic process (Table 3). Given the time course of symptom development over weeks, in conjunction with these electrodiagnostic features, the diagnosis of the AMSAN variant of Guillain-Barré syndrome was suggested. Treatment with 2 g/kg intravenous immunoglobulin (IVIg) was administered over five doses while inpatient.

**Table 1:** Motor Nerve Conduction Study - Anti Abbreviations: APB = abductor pollicis brevis, EDB = extensor pollicis brevis, AH = abductor hallucis, Tib Ant = tibialis anterior, Ref = reference, Lat diff = latency difference, NR = no response, Pop fossa = popliteal fossa, L = Left, R = Right

Nerve/Sites	Muscle	Latency (ms)	Ref (ms)	Amplitude (mV)	Ref. (mV)	Amp (%)	Duration (ms)	Segments	Distance (mm)	Lat Diff (ms)	Velocity (m/s)	Ref. (m/s)	Area (mVms)
R Median - APB													
Wrist	APB	4.6	≤4.2	0.4	≥3.5	100	8.9	Wrist - APB	70				1.8
Elbow	APB	10.6		0.3		87.7	9.9	Elbow - Wrist	245	6	41	≥48	1.6
L Median - APB													
Wrist	APB	4.9	≤4.2	1.4				Wrist - APB					5.4
Elbow	APB	11.8		1.2		87	7	Elbow-wrist	245	6.8	36	≥48	4.5
R Peroneal - EDB	EDB												
Ankle	EDB	NR	≤5.5	NR		NR	NR	Ankle - EDB	70				NR
R Tibial - AH													
Ankle	AH	12	≤6.0	0.1	≥2.9	100		Ankle - AH	100				
R Peroneal - Tib Ant													
Fib Head	Tib Ant	4.1	≤6.7	1.8	≥3.0	100	13.6	Fib Head - Tib Ant	90			≥44	15.1
Popliteal Fossa	Tib Ant	6.4		1.5		86.4	13.9	Pop fossa - Fib Head	120	2.3	52		14.3

**Table 2:** Sensory Nerve Conduction Study - Anti Abbreviations: Onset lat = onset latency, ref = reference, B-P Amp = baseline to peak amplitude, P-P Amp = peak to peak amplitude, NR = no response, Dig = digit, L = Left, R = Right

Nerve/Sites	Rec. Site	Onset Lat (ms)	Ref. (ms)	B-P Amp (μV)	Ref. (μV)	P-P Amp micron	Ref. (μV)	Segments	Distance (mm)	Velocity (m/s)	Ref. (m/s)
R Ulnar - Digit V											
Wrist	Div V	NR	≤3.1	NR	≥15.0	NR		Wrist-Dig V	140	NR	≥45
L Ulnar - Digit V											
Wrist	Dig V	NR	≤3.1	NR	≥15.0	NR		Wrist-Dig V	140	NR	≥45
R Median - Digit II											
Wrist	Dig II	NR	≤3.5	NR	≥19.0	NR		Wrist - Dig II	140	NR	≥45
L Median - Digit II											
Wrist	Dig II	NR	≤3.5	NR	≥19.0	NR		Wrist - Dig II	140	NR	≥45
R Radial - Anatomical Snuff Box (Forearm)											
forearm	wrist	NR	≤2.0	NR	≥15.0	NR	≥15.0	Forearm-Wrist	100	NR	≥50
R Sural - Ankle (Calf)											
Calf	Ankle	NR	≤4.4	NR	≥5.0	NR		Calf-Ankle	140	NR	≥38

**Table 3:** Electromyography Abbreviations: MUAP = motor unit action potential, N = normal, R. = right, L. = left, IA = insertion activity, Fib= fibrillations, PSW = poly spike wave, Fasc = fasciculations, Amp = amplitude, Dur = duration, PPP = polyphasic potential, CRDs = complex repetitive discharges

	Spontaneous					MUAP			Recruitment Pattern	Interference Pattern	Firing Rate
Muscle	IA	Fib	PSW	Fasc	Other	Amp	Dur	PPP			
R. Tibialis Anterior	N	2+	2+	1+	None	1+	1+	1+	Decreased	Mixed	Increased
L. Gastrocnemius (medial head)	N	2+	2+	1+	None	N	1+	1+	Decreased	Mixed	Increased
R. Rectus Femoris	N	None	None	1+	CRDs	1+	N	2+	Decreased	Mixed	Increased

During the hospitalization, the patient experienced a nadir in her weakness, with 1-2/5 strength in the right upper extremity and 2-3/5 in the left upper extremity with scarce fasciculations present and generalized muscle wasting. She was areflexic throughout. Sensation to vibration was markedly reduced distal to the knees and in the fingertips, and sensation to pinprick remained intact. The patient was discharged to a rehabilitation facility where intensive physical therapy was pursued. Over the course of approximately three months, the patient’s strength improved to 4/5 in upper and lower extremities bilaterally with symmetrically persistent weakness in finger abduction bilaterally. Vibration sense also improved. At the three-month follow-up, the patient was not yet ambulating independently but was able to perform transitions with help from physical therapy. At five months post-discharge, the patient was able to walk long distances with a walker, and was able to walk up and down stairs. 

## Discussion

AMSAN is a variant of Guillain-Barré syndrome and is caused by immune-mediated damage to the axons of myelinated sensory and motor nerves. It typically presents with a progressive, symmetric muscle weakness, decreased sensation, and absent deep tendon reflexes as patients approach the nadir of their disease progression. These symptoms classically occur over a period of days to weeks, with the majority of patients reaching the nadir of disease by four weeks. Our patient exhibited all of these findings and reached nadir at approximately two weeks after symptom onset. The clinical diagnosis of AMSAN can be supported by cerebrospinal fluid analysis showing albuminoctyologic dissociation and electro-diagnostic testing showing reduced/absent compound muscle action potential and sensory nerve action potential amplitudes. Additionally, nerve root enhancement is often seen on magnetic resonance imaging of the lumbar spine in patients with acute inflammatory demyelinating polyneuropathy. However, not all patients demonstrate this classic nerve root enhancement. Further, one study has shown that variants of Guillain-Barré syndrome demonstrate a lesser incidence of root enhancement when compared to the classical type [8]. Additionally, studies in pediatric populations of shown that there is variable appearance of the root enhancement when it is present [9]. Currently, there are no consensus criteria for establishing the diagnosis of AMSAN.

Treatment involves either IVIg or plasma exchange depending on institutional availability and patient-specific risk factors. Both treatments improve patient outcome, however plasma exchange has been associated with higher rates of complications [10]. However, treatment with either modality does not guarantee a complete recovery, as a minority of patients experience a prolonged disease course with incomplete recovery.

Supportive treatment is imperative, as patients are at risk for developing respiratory failure requiring mechanical ventilation and/or autonomic dysfunction requiring intensive care unit-level care. Thus, these patients should be monitored with frequent neurologic exams with muscle strength testing in all extremities as well as facial/bulbar/neck strength, as weakness in those muscle groups portends worse prognosis and possible need for ventilatory support. Hemodynamic monitoring as well as ventilation status assessment with oxygen saturation, respiratory rate, negative inspiratory force and forced vital capacity should also be monitored closely to assess signs of respiratory failure.

This case highlights the possibility of investigational antibody-drug conjugates as a potential cause of AMSAN. It is possible that certain drug-antibody conjugates could cross-react with epitopes present on the peripheral nerve. While the complete spectrum of potential causes of AMSAN remains an area of incomplete knowledge, there have been reported cases of AMSAN with polychemotherapy regimens [3] as well as tumor necrosis factor alpha antagonist therapy [11]. Additionally, our patient was likely at increased risk of developing a sensorimotor polyneuropathy due to multiple vitamin B deficiencies. While our patient received one five-day course of IVIg and experienced improvement over the course of months, the duration of treatment with IVIg is not well-defined in this situation, especially without a clear underlying antibody and with subacute features on nerve conduction studies. Further, the patient’s rapid improvement of motor strength suggests that conduction block may have contributed to her profound weakness and electrodiagnostic changes, as the time course of axonal regeneration can be unpredictable, but typically occurs over a period of six to 12 months.

## Conclusions

This case report concludes that novel antibody-drug conjugates can potentially induce AMSAN, and treatment with IVIg can be beneficial. It is important to consider other reversible patient conditions that could lead to neuropathy, such as B vitamin deficiencies, which may be superimposed and can confound the clinical picture. Further studies are needed to elucidate the exact mechanism of AMSAN due to antibody-drug conjugates, as well as the best course and duration of treatment. 
